# Hypertension and Maternal–Fetal Conflict during Placental Malaria

**DOI:** 10.1371/journal.pmed.0030446

**Published:** 2006-11-14

**Authors:** Atis Muehlenbachs, Theonest K Mutabingwa, Sally Edmonds, Michal Fried, Patrick E Duffy

**Affiliations:** 1 MOMS Project, Seattle Biomedical Research Institute, Seattle, Washington, United States of America; 2 University of Washington, Seattle, Washington, United States of America; 3 London School of Hygiene and Tropical Medicine, London, United Kingdom; 4 National Institute for Medical Research, Dar es Salaam, Tanzania; 5 Muheza Designated District Hospital, Muheza, Tanzania; 6 Walter Reed Army Institute of Research, Silver Spring, Maryland, United States of America; Rigshospitalet, Denmark

## Abstract

**Background:**

Malaria and hypertension are major causes of maternal mortality in tropical countries, especially during first pregnancies, but evidence for a relationship between these syndromes is contradictory.

**Methods and Findings:**

In a cross-sectional survey of Tanzanian parturients, the rate of hypertension was similar in placental malaria (PM)-positive (11/85 = 13%) and PM-negative (73/602 = 12%) individuals. However, we found that PM was associated with hypertension in first-time mothers aged 18–20 y but not other mothers. Hypertension was also associated with histologic features of chronic malaria, which is common in first-time mothers. Levels of soluble vascular endothelial growth factor receptor 1 (sVEGFR1), a preeclampsia biomarker, were elevated in first-time mothers with either PM, hypertension, or both, but levels were not elevated in other mothers with these conditions. In first-time mothers with PM, the inflammatory mediator vascular endothelial growth factor (VEGF) was localized to maternal macrophages in the placenta, while sVEGFR1, its soluble inhibitor, was localized to the fetal trophoblast.

**Conclusions:**

The data suggest that maternal–fetal conflict involving the VEGF pathway occurs during PM, and that sVEGFR1 may be involved in the relationship between chronic PM and hypertension in first-time mothers. Because placental inflammation causes poor fetal outcomes, we hypothesize that fetal mechanisms that promote sVEGFR1 expression may be under selective pressure during first pregnancies in malaria-endemic areas.

## Abstract 2

Children of mothers with placental malaria were more likely to exhibit parasitemia within their first year. First-born children were at a lower risk, regardless of whether their mothers had placental malaria.

## Editors' Summary

**Background.:**

Malaria is one of the world's most serious health problems. It causes about 1 million deaths every year, and most of these deaths are in children. Several different parasites can cause malaria; the most serious is Plasmodium falciparum. One of the most serious consequences of infection is that this parasite can multiply in the placenta of a pregnant woman. This “placental malaria” is very harmful to the mother and to the fetus; it leads to low birth weight and is estimated to be responsible for the deaths every year of about 200,000 babies within their first year of life. A woman who is pregnant for the first time is most likely to suffer from placental malaria, and to have her placenta become highly infected and extremely inflamed. If she later becomes pregnant again, she will be protected to some extent by antibodies she has developed against the parasite.

Another problem that is common in tropical countries and also causes many deaths during pregnancy is preeclampsia—high blood pressure (hypertension) and protein loss in the urine. This is also a condition that is most common in first-time mothers. The causes of preeclampsia are not clear, but many factors are probably involved. Among the theories that have been proposed are that inflammation in the placenta might play a part, and that there may be a “conflict” between the needs of the mother and those of the fetus.

**Why Was This Study Done?:**

The researchers wanted to see whether placental malaria might be a factor in the development of preeclampsia. This association has been suggested before, but there has been no clear evidence.

**What Did the Researchers Do and Find?:**

Working with pregnant women in Tanzania, they found that, overall, women with placental malaria were no more likely than other women to develop hypertension. However, for those women who were aged 18–20 and pregnant for the first time, having placental malaria was associated with hypertension. The researchers also measured levels of a substance called sVEGFR1 (also called sFlt1), which is known to increase before and during preeclampsia and is thus considered to be a biomarker for the condition. sVEGFR1 levels were high in first-time mothers with either placental malaria or hypertension, or both, but levels were not raised in other mothers with these conditions. A related substance, VEGF, which is known to be involved with the process that causes inflammation, was high in first-time mothers with placental malaria, but not in those who had preeclampsia alone.

**What Do These Findings Mean?:**

The researchers believe that their findings support the view that, in younger first-time mothers only, placental malaria can cause preeclampsia and that this results from a conflict between the mother and her fetus. Action to reduce the chance of such women getting malaria would have the additional benefit of lowering their chance of developing preeclampsia. The findings have also led the researchers to propose possible mechanisms as to how placental malaria leads to preeclampsia. They have made suggestions regarding the further research that is now needed.

**Additional Information.:**

Please access these Web sites via the online version of this summary at http://dx.doi.org/10.1371/journal.pmed.0030446.

- Wikipedia, an online encyclopedia that anyone can edit, has pages on malaria
- The World Health Organization (WHO)
- UNICEF (the United Nations children's organization)
- MedlinePlus brings together authoritative information from the US National Library of Medicine, National Institutes of Health, and other government agencies and health-related organizations.
- The Wellcome Trust

## Introduction

Placental malaria (PM) caused by Plasmodium falciparum preferentially affects first-time mothers: rates of up to 70% occur in regions of sub-Saharan Africa, where low birth weight due to PM is estimated to cause 200,000 infant deaths each year. Among the dozens of malaria parasite species that infect mammals, only P. falciparum is known to sequester in the placenta during naturally occurring infections [1]. During PM, infected erythrocytes (IE) adhere to chondroitin sulfate A and sequester in the intervillous spaces of the placenta [2]. Over successive pregnancies, women acquire antibodies that block IE adherence [3]. First-time mothers lack these antibodies, and therefore often experience dense parasitemias and intense inflammatory responses.

Hypertensive disorders of pregnancy occur primarily in humans, and are estimated to cause 10%–15% of maternal deaths [4]. Preeclampsia, defined by hypertension and proteinuria, is the most well-described disorder [5]. Like PM, preeclampsia is more common in first-time mothers, but for unknown reasons. The pathogenesis of preeclampsia is obscure, although abnormal placentation is currently considered among the more plausible hypotheses. Recent findings and theories reviewed elsewhere [5–8] have variously imputed roles for systemic inflammation, abnormalities in cardiovascular adaptation to pregnancy, agonistic autoantibodies to the angiotensin receptor, and aberrations in calcium metabolism, as well as anti-angiogenic factors (discussed further below). Fetal mechanisms that cause hypertension have been proposed to have evolved as a result of maternal–fetal conflict over nutrient allocation [9,10].

Malaria is well known to decrease blood pressure in non-pregnant individuals, but the relationship between PM and blood pressure is not clear [11]. During the 1935 malaria epidemic in Sri Lanka, the obstetrician Wickramasuriya described an “‘epidemic' of … toxaemic pregnancies following in the wake of the malaria epidemic”, with hypertension in 20%, albuminuria in 40%, edema in 50%, and death in 13% of 357 infected women [12]. In sub-Saharan Africa, the rates of both preeclampsia and malaria increase during the cooler rainy season [13–16]; however, in non-malarious areas preeclampsia may also increase during colder months [17]. Preeclampsia increased the odds of PM in Senegal [15]; however, it was not associated with peripheral parasitemia [18] or with placental infection by histology [19] in Kenya.

Serum levels of placentally derived soluble vascular endothelial growth factor receptor 1 (sVEGFR1) are elevated prior to [20] and during [21] preeclampsia. (sVEGFR1 is also called soluble fms-like tyrosine kinase 1 [sFlt1].) sVEGFR1 may cause systemic endothelial dysfunction by binding and sequestering free serum vascular endothelial growth factor (VEGF) and placental growth factor [20,21]. In earlier studies, hypertension, proteinuria, and glomerular endotheliosis developed in rats infected with a recombinant retrovirus encoding sVEGFR1 [21], and hypertension and proteinuria developed in cancer patients receiving monoclonal anti-VEGF therapy [22]. Astrocyte VEGF expression and extracellular VEGFR1 have been observed in the brain of cerebral malaria patients [23], but have not been examined in other malaria syndromes.

## Methods

### Human Participants

Placental samples and clinical information were provided by Tanzanian women aged 18–45 y delivering at the Muheza Designated District Hospital, Muheza, Tanga region. Sample donors were among those recruited to participate in a birth cohort study known locally as the Mother-Offspring Malaria Study. Women provided signed informed consent before joining the study, and those with chronic debilitating disease and their children were excluded. Clinical information was collected by project nurses and assistant medical officers on standardized forms. Study procedures involving human participants were approved by the International Clinical Studies Review Committee of the Division of Microbiology and Infectious Diseases at the US National Institutes of Health, and ethical clearance was obtained from the Institutional Review Board of Seattle Biomedical Research Institute and the National Medical Research Coordinating Committee in Tanzania.

### Study Procedures

Hypertension was defined as systolic or diastolic blood pressure greater than or equal to 140 mm Hg or 90 mm Hg, respectively. Blood pressure was abstracted from hospital delivery cards as the maximum measurement recorded during the delivery hospitalization. Blood pressure measurements were not taken during active labor. Urine protein assessment was not routinely performed in the hospital. Immediately prior to delivery, peripheral blood was collected in citrate phosphate dextrose, and plasma was separated and frozen at −80 °C. The placenta was collected at delivery, and a full thickness biopsy from the middle third of the placental disc was frozen in liquid nitrogen and stored at −80 °C. PM was detected by microscopy of Giemsa-stained thick and thin smears of placental blood extracted from placental tissue by mechanical grinding. Placental parasite density was quantified as percent IE.

### Histopathologic Analysis

Cryosections (5 μm) of placental tissue were Giemsa stained and assessed by examining greater than 90 60X fields per section. Malarial pigment is a brown heme-derived product that refracts under polarizing light and persists in acellular fibrinoid in the intervillous space. Pigment deposition in fibrinoid was quantified by determining the proportion of fields with pigment present. Inflammation was qualitatively scored by the presence of inflammatory cells in the intervillous space.

### Enzyme-Linked Immunosorbent Assay

Soluble VEGFR1 levels in peripheral plasma were determined in duplicate by ELISA kit DVR100A (R&D Systems, Minneapolis, Minnesota, United States). Levels were corrected for dilution volume in anticoagulant, and over-diluted samples were detected by low potassium concentration as measured by EasyLyte Plus (Medica, Bedford, Massachusetts, United States) and excluded.

### Quantitative RT-PCR

Total RNA was extracted from frozen cryosections using RNeasy minikits (Qiagen, Hilden, Germany). RNA quality was assessed by Agilent 2100 Bioanalyzer (Santa Clara, California, United States), resulting in 28/18s ratios of 1.1 to 1.5. cDNA was synthesized using Superscript III enzyme (Invitrogen, Carlsbad, California, United States) and anchored oligodT20 primers. Exon-spanning primers were designed for sVEGFR1:GGGGAAGAAATCCTCCAGAA(fwd), AGCCTTTTTGTTGCAGTGCT(rev), (63-bp product); VEGF (all isoforms): CCCACTGAGGAGTCCAACAT(fwd), TGCATTCACATTTGTTGTGC(rev), (106-bp product); and cytokeratin 7: GGCTGAGATCGACAACATCA(fwd), CTTGGCACGAGCATCCTT(rev) (103-bp product). Real-time PCR was performed in duplicate using SYBR green master mix and an ABI Prism 7000 (Applied Biosystems, Foster City, California, United States) with an annealing temperature of 60 °C. Threshold cycles (C_T_) were normalized to C_T_ of KRT7, and *t*-tests performed on normalized C_T_ values. Data are presented as fold-difference from control gene, calculated by log_2_^(normalized CT)^.

### Immunostaining

Cryosections (5 μm) were fixed in paraformaldehyde, blocked in chicken and goat sera (Santa Cruz Biotechnology, Santa Cruz, California, United States, and Amersham, Little Chalfont, United Kingdom, respectively), and variously probed with mouse anti-VEGFR1 extracellular domain (R&D Systems) at 1:400, which detects both the membrane-bound and soluble isoforms of VEGFR1, mouse anti-VEGF clone VG1 (Chemicon, Temecula, California, United States) at 1:200 and rabbit anti-placental lactogen (Dako, Glostrup, Denmark) at 1:1,000 dilution. Secondary antibodies included Alexafluor 488 chicken anti-mouse antibody (Molecular Probes, Eugene, Oregon, United States) and TRITC goat anti rabbit antibody (Sigma, St. Louis, Missouri, United States). DAPI (Sigma) was used to define nuclear DNA. VEGF was also visualized using DAB Envision + kit (Dako), and sections were counterstained with Giemsa.

### Statistical Analysis

Analyses were performed using Statview (SAS Institute, Cary, North Carolina, United States). Student's *t*-test was used for the analysis of continuous variables. Mann-Whitney test was used to examine parity and placental pigment deposition. Peripheral plasma sVEGFR1 concentration and parasite density (n + 1) were log-transformed prior to analysis. Odds ratios were calculated using logistic-regression analysis. Regression coefficients were calculated using simple regression analysis. Interactions between variables were determined using factorial ANOVA. Spearman rank correlation was used to examine the correlation between sVEGFR1 quantitative PCR and ELISA measurements.

## Results

We examined the relationship between PM and hypertension in northeastern Tanzania. The analysis protocol for the study was to examine the relationship of demographic factors including parity and age to hypertension in women stratified by PM status. In first-time mothers, who are at risk for both PM and preeclampsia, we examined the histological features of PM that were associated with hypertension. We examined the expression of sVEGFR1 in women stratified by parity, PM status, and hypertension.

Placental samples and clinical information were provided by women delivering at the Muheza Designated District Hospital, in an area of intense malaria transmission [24]. Analyses included 887 women with viable deliveries who joined the study between September 2002 and March 2005. Twins were excluded. Blood pressure data were available for 688 women. Women without blood pressure measurements did not differ in malaria status, age, or parity, but had a mean of 70-g (95% confidence interval [CI] [1–150g]) smaller babies (Table S1). As previously observed, PM was associated with first pregnancy, younger age, and lower birth weight (Table 1).

**Table 1:**
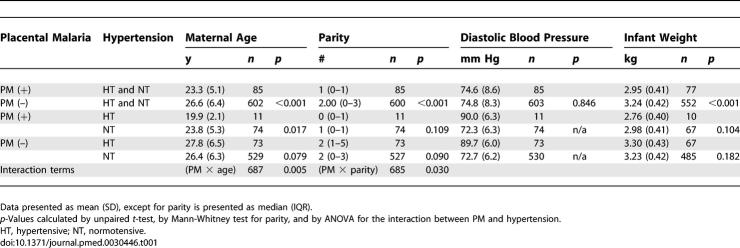
Clinical Data for the Women in the Study, Stratified by PM Status and Hypertension

**Figure 1:**
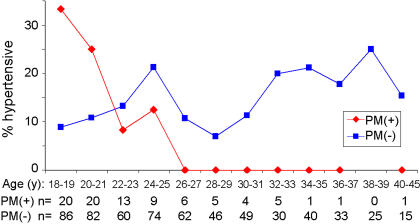
Line Graph Illustrating the Prevalence of Hypertension in PM-Positive and PM-Negative Women in Different Age Strata

When women of all ages were analyzed in aggregate, blood pressure did not differ between PM-positive and PM-negative women (Table 1), consistent with previous reports. Because age and parity are independently related to risk of both PM and hypertension, we examined the effects of age and parity on the relationship between PM and hypertension (Table 1). Hypertensive PM-positive women were significantly younger than normotensive PM-positive women, and also had a trend toward lower parity (Table 1). When hypertension prevalence was examined over different age strata, PM appeared to increase the risk of hypertension in younger women, but decreased the risk in older women (Figure 1). By factorial ANOVA, the interaction term (age × PM) significantly modified the relationship between PM and hypertension (*p* = 0.005). The interaction term (parity × PM) had a similar effect that was also significant (*p* = 0.030). When data were stratified by parity and median age of first-time mothers, PM-positive women were at significantly increased odds of hypertension compared with PM-negative women for first-time mothers aged 18–20 y (odds ratio [OR] [95% CI] = 3.1 [1.1–9.0], *n* = 142, *p* = 0.035), but not older first-time mothers (zero cases, *n* = 60), or mothers of later pregnancy (OR [95% CI] = 0.6 [0.2–1.9], *n* = 483, *p* = 0.418).

We examined placental pigment deposition and placental parasite density in PM-positive women to determine whether chronic forms of PM were associated with hypertension (Table 2). As previously observed, pigment deposition was greatest in first-time mothers, and was negatively correlated with birth weight (R = −0.525, *n* = 32, *p* = 0.002). PM-positive first-time mothers with hypertension had significantly increased amounts of pigment compared with PM-positive first-time mothers without hypertension. Hypertension was associated with low placental parasite density, particularly in first-time mothers. Placental parasite density was negatively associated with diastolic blood pressure (all mothers: R= −0.212, *p* = 0.052, *n* = 85; first-time mothers: R = −0.345, *p* = 0.034, *n* = 38). Thus, hypertension in PM-positive women was associated with features of chronic disease, such as heavier pigment deposition and lower parasite density.

**Table 2:**
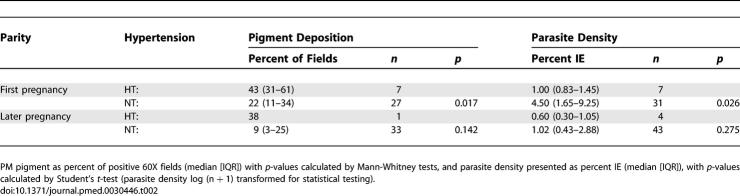
Relationship between Hypertension and Placental Parasite Density and Malarial Pigment in PM-Positive Women

**Figure 2:**
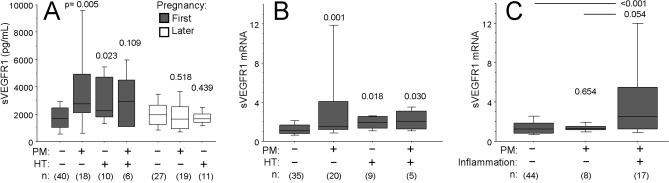
sVEGFR1 Expression Levels in PM-Positive and PM-Negative Women (A) Peripheral plasma sVEGFR1 levels (median [IQR]). Levels are indicated for mothers stratified by parity, PM, and hypertension (HT). (B and C) Placental sVEGFR1 mRNA abundance (median [IQR]) in first-time mothers. Levels indicate fold-increase over trophoblast-specific cytokeratin 7 (KRT7) mRNA. Women were stratified (B) by PM and hypertension or (C) by PM and intervillous inflammation.

We measured sVEGFR1 levels in peripheral plasma by ELISA (Figure 2A). sVEGFR1 levels in PM-negative normotensive first-time mothers were comparable to levels in healthy first-time mothers from non-endemic countries [20]. Compared with uninfected first-time mothers, sVEGFR1 levels were significantly elevated in first-time mothers with PM or with hypertension, and were non-significantly elevated in those with both. During later pregnancies, sVEGFR1 levels did not differ with PM. We determined sVEGFR1 transcript abundance in placental tissue of first-time mothers by quantitative RT-PCR (Figure 2B). Placental transcript levels correlated with peripheral protein levels (Pearson Rho = 0.483, *p* < 0.001, *n* = 49), and were significantly elevated in first-time mothers with PM, hypertension, or both. sVEGFR1 transcript levels were specifically elevated in tissue that had maternal inflammatory cells in the intervillous space (Figure 2C).

**Figure 3:**
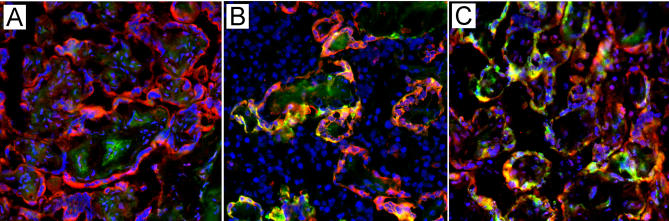
Immunofluorescence of Placental Cryosections from First-Time Mothers Showing VEGFR1 Extracellular Domain (Green), Trophoblast (Red), and Nuclear DNA (Blue) All fields are 200X magnification. Cryosections from (A) PM-negative normotensive pregnancy; (B) PM-positive normotensive pregnancy with intervillous inflammation; (C) PM-positive hypertensive pregnancy.

We explored whether sVEGFR1 expression occurred in villous trophoblast cells, which are of fetal origin (Figure 3). In uninfected placentas, VEGFR1 immunoreactivity in villous trophoblast was not observed. In infected placentas from first-time mothers who were hypertensive or had inflammatory infiltrates, VEGFR1 immunoreactivity co-localized with trophoblast, and not with maternal inflammatory cells.

**Figure 4:**
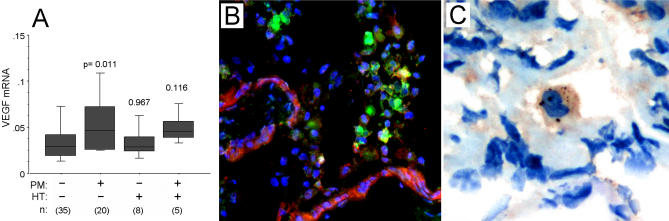
Placental VEGF Expression and Localization in First-Time Mothers (A) Placental VEGF mRNA abundance (median [IQR]) in first-time mothers. Levels indicate fold-increase over trophoblast-specific cytokeratin 7 (KRT7) mRNA. (B and C) Visualization of VEGF in placental cryosections from first-time mothers with intervillous inflammation (B) by immunofluorescence with VEGF (green), trophoblast (red), and nuclear DNA (blue) labeling, 320X magnification, and (C) by immunohistochemistry using DAB and counterstained with Giemsa, 400X magnification.

Placental transcript levels of VEGF, a ligand for sVEGFR1, were also significantly elevated in first-time mothers with PM, but were not elevated in those with hypertension alone (Figure 4A). In contrast to the VEGFR1 localization studies, maternal inflammatory cells but not trophoblast cells were immunoreactive for VEGF in first-time mothers with PM (Figure 4B). VEGF-reactive cells had the morphology of pigment-containing macrophages (Figure 4C).

## Discussion

In this study, PM was associated with hypertension in young first-time mothers with histologic features of chronic disease, but was not related to hypertension in older or multigravid women. Similarly, PM was associated with elevated levels of sVEGFR1 in first-time mothers but not in later mothers. In first-time mothers with PM, sVEGFR1 was localized to the fetal trophoblast and VEGF was localized to maternal macrophages. Limitations of this study included the absence of proteinuria data, preventing the differential diagnosis between preeclampsia and gestational hypertension, and the lack of longitudinal or interventional data.

We propose a model in which chronic PM during first pregnancies causes hypertension. The effect of chronic PM to increase blood pressure may be mediated by trophoblast expression of sVEGFR1. Malaria infection lowers blood pressure in non-pregnant individuals, and in our study placental parasite density was negatively associated with blood pressure in first-time mothers. Some first-time mothers with PM and elevated sVEGFR were normotensive, and we speculate that the hypotensive effects of heavy parasite density may sometimes counteract the hypertensive effects of sVEGFR1, or, alternatively, that VEGF at high levels in some women may exceed the binding capacity of sVEGFR1 and prevent hypertension. A hypotensive effect of parasite density may be mediated directly by the parasite as observed with dog heartworm [25], or indirectly by host responses such as elevated nitric oxide levels.

sVEGFR1 levels are proposed to have utility for detecting preeclampsia. Our data indicate that such a test would be confounded by PM in malarious areas, because normotensive first-time mothers with PM had the highest sVEGFR1 levels. However, sVEGFR1 levels may have utility in identifying PM-positive women in need of treatment, given the low specificity (~50%) of peripheral blood smear diagnosis for detecting PM. Further, we do not know whether the normotensive women with elevated sVEGFR1 levels would have gone on to develop hypertension if they had not delivered.

We observed VEGFR1 localization in the trophoblast but not in the maternal inflammatory cells. Trophoblast VEGFR1 expression has been reported [26,27]; however, as a cautionary note, one study suggested that maternal immune cells contribute to sVEGFR1 levels in preeclampsia [28]. We observed VEGF expression in maternal macrophages, but not the villous trophoblast. Macrophages have previously been reported to be a source of VEGF [29,30].

The role of sVEGFR1 in healthy pregnancy is not known; however, VEGF administration in one study caused pregnancy loss in mice [31]. VEGF induces monocyte activation and chemotaxis [32–34], which can be blocked experimentally by sVEGFR1 [34,35]. We speculate that the fetus expresses sVEGFR1 during PM in an effort to limit the maternal inflammatory response, and therefore increased sVEGFR1 production may be under selective pressure in malaria-endemic areas, particularly during first pregnancies. We further hypothesize that exposure to P. falciparum malaria during human history may have contributed to the elevated sVEGFR1 levels observed in healthy first versus second pregnancies [36], and the increased prevalence of preeclampsia observed among people of African [37–39] or South Asian [40,41] ancestry, and may also explain why preeclampsia is primarily a human condition.

In conclusion, hypertension occurs in young first-time mothers with chronic malaria, and their elevated sVEGFR1 levels suggest they are suffering from preeclampsia. Clinical studies should address the relationship between peripheral parasitemia, sVEGFR1 levels, and blood pressure longitudinally over the course of gestation, and should examine the effects of VEGFR1 gene polymorphisms on these outcomes. Effective prevention of PM could lower the incidence of hypertension and should be examined in interventional trials.

## Supporting Information

Table S1 Clinical Data for Women Recruited into the MOMS Project, Stratified by Whether or Not Blood Pressure Measurements Were Recorded (28 KB DOC)

## Abbreviations

CI: confidence interval
IE: infected erythrocyte
OR: odds ratio
PM: placental malaria
sVEGFR1: soluble vascular endothelial growth factor receptor 1
VEGF: vascular endothelial growth factor
