# Supplementary material for: Hypertension and Maternal–Fetal Conflict during Placental Malaria
Source: PLoS Med. 2006 Nov 14;3(11):e446. doi: 10.1371/journal.pmed.0030446 (PMC1635741; doi:10.1371/journal.pmed.0030446)
Supplement: Supplementary file 1 [file pmed.0030446.st001.doc]

Table S1. Clinical data for women recruited into the MOMS Project, stratified by whether or not blood pressure measurements were recorded.

|  | PM rate | | | Age | | | Parity | | | Infant weight | | |
| --- | --- | --- | --- | --- | --- | --- | --- | --- | --- | --- | --- | --- |
| Blood pressure record | % | n | p | years | n | p | # | n | p | kg | n | p |
| Recorded | 12.3 | 85/688 |  | 26.2 (6.3) | 687 |  | 1.87 (1.85) | 685 |  | 3.20 (0.43) | 629 |  |
| Not recorded | 10.5 | 21/199 | 0.476 | 25.7 (6.2) | 197 | 0.329 | 1.72 (1.84) | 197 | 0.377 | 3.13 (0.42) | 183 | 0.023 |

PM rate is reported as percent, all other data as mean (SD). p-values were calculated using Chi-squared test for rate of infection, all others using unpaired t-tests. BP= blood pressure.
